# Efficacy of Intra-arterial [^177^Lu]Lu-DOTATATE monotherapy for treatment-refractory meningioma

**DOI:** 10.1007/s11060-025-05266-9

**Published:** 2025-10-15

**Authors:** A. El Ghalbouni, T. J. Snijders, A. Amerein, N. Tolboom, M. Patt, C. J. Maurer, I. C. van der Schaaf, C. Lapa, A. J. A. T. Braat

**Affiliations:** 1 Radiology and Nuclear Medicine, UMC Utrecht, Heidelberglaan 100, Utrecht, 3584 CX Netherlands; 2 Neurology and Neurosurgery, Brain Center, UMC Utrecht, Utrecht, The Netherlands; 3 Nuclear Medicine, Faculty of Medicine, University of Augsburg, Augsburg, Germany; 4 Diagnostic and Interventional Neuroradiology, Faculty of Medicine, University of Augsburg, Augsburg, Germany; 5 Nuclear Medicine, Netherlands Cancer Institute, Amsterdam, The Netherlands

**Keywords:** Meningioma, Treatment-refractory, Efficacy, Safety, Intra-arterial, [^177^Lu]Lu-DOTATATE

## Abstract

**Purpose:**

For meningiomas that are refractory after local treatments, there are no systemic evidence-based therapeutic options to date. In recent years, somatostatin-targeted radionuclide therapy has emerged as a promising new treatment modality. Recent studies have indicated that intra-arterial administration of [^177^Lu]Lu-DOTATATE may increase the tumor absorbed dose. This study aims to assess the efficacy of intra-arterial [^177^Lu]Lu-DOTATATE in a cohort of treatment-refractory meningiomas.

**Materials and methods:**

Retrospective cohort study including patients diagnosed with treatment-refractory meningioma (WHO grade 1–3), who had received at least one cycle of intra-arterial [^177^Lu]Lu-DOTATATE. Primary outcomes were progression-free survival (PFS) and overall survival (OS). Secondary outcomes included objective response, and safety and toxicity.

**Results:**

Seventeen patients with a median follow-up duration of 36 months (range: 1–57 months) were included. Six-month PFS was 65% (95% CI: 46–92%) and 6-month OS was 82% (95% CI: 66–100%). Regarding best objective response, disease control was found in 53% (95% CI: 28–77%); with an objective response rate of 24% (95% CI: 7–50%). In terms of safety, two patients developed grade 3 anemia, while one patient experienced local necrosis as a result of peripheral embolism, a complication related to angiographic intervention.

**Conclusion:**

In a treatment-refractory meningioma cohort, intra-arterial administration of [^177^Lu]Lu-DOTATATE is safe and effective, with an objective response rate of 24%, and survival data that exceed published benchmarks. Prospective controlled studies with larger cohorts and extended follow-up are needed, comparing intra-arterial [^177^Lu]Lu-DOTATATE to (historic) cohorts receiving intravenous administration are needed.

**Supplementary Information:**

The online version contains supplementary material available at 10.1007/s11060-025-05266-9.

## Introduction

Meningiomas represent the most prevalent primary nonglial intracranial tumors, exhibiting an incidence of 10 per 100,000 individuals [1]. According to the World Health Organization (WHO) classification of central nervous system tumors, meningiomas are categorized into three grades based on histological characteristics: benign (grade 1), atypical (grade 2), and anaplastic (grade 3). In the recent cIMPACT-NOW 8 recommendations, molecular markers were added to histology to refine classification and prognostication [2]. A subset of meningiomas, mostly higher-grade lesions, exhibits aggressive clinical behavior with frequent recurrence after local treatments such as surgery or external beam radiotherapy (EBRT). The risk of recurrence is also increased in anatomically challenging regions, such as the cavernous sinus or optic nerve sheath, where achieving gross total resection is often not feasible and re-irradiation poses significant toxicity risks. For these treatment-refractory meningiomas, no evidence-based treatment protocol exists. Therapeutic options are limited, with patients often subjected to experimental systemic interventions [3–6].

Overexpression of the somatostatin receptor subtype 2 (SSTR2) on meningiomas has been broadly confirmed [7]. SSTR2-targeted radionuclide therapy, commonly referred to as ‘peptide receptor radionuclide therapy’ (PRRT) has been acknowledged by the European Association of Neuro-oncology (EANO) as a salvage therapy for treatment-refractory meningioma [3], as outlined in recent practice guidelines [8].

Most studies have assessed the efficacy of PRRT through intravenous administration, predominantly with [^177^Lu]Lu-DOTATATE monotherapy, reporting a 6-month progression-free survival (PFS6) rate of approximately 60% [9, 10]. The largest prospective study, which included 42 patients and had a median follow-up of 63 months, reported a disease control rate (DCR) of 57% [11]. It is hypothesized that the efficacy of [^177^Lu]Lu-DOTATATE correlates with the radiation dose absorbed by the tumor. Previous research on intra-arterial administration for neuroendocrine tumors (NETs) [12], along with recent case series on meningiomas [13, 14], suggests that intra-arterial administration enhances tumor absorbed dose, potentially increasing efficacy. This study, therefore, aims to evaluate the efficacy and safety of intra-arterial [^177^Lu]Lu-DOTATATE in treatment-refractory meningiomas.

## Methods

### Patients

This retrospective cohort study was conducted in two centers. Patients were included if they met the following criteria: 1) ≥ 18 years of age, 2) having treatment-refractory meningioma WHO grade 1–3, 3) had undergone at least 1 intra-arterial administered cycle of [^177^Lu]Lu-DOTATATE. Informed consent was waived by both institutional ethics committees, because of the retrospective nature of this study. Collected data included: age at diagnosis, age at first cycle, number of intravenous and intra-arterial cycles, cumulative administered dose (in GBq), target artery, WHO grade, tumor location, Eastern Cooperative Oncology Group (ECOG) performance status, neurological deficits, prior interventions and tumor volume on T1 gadolinium-enhanced MRI. For cases involving multifocal meningiomas, the largest lesion was designated as the target lesion.

### Eligibility

All patients with progressive meningioma were reviewed by a multidisciplinary tumor board and were considered treatment-refractory if surgery or EBRT were no longer viable options. [^177^Lu]Lu-DOTATATE was administered as routine clinical care. Treatment eligibility was according to the EANM/EANO/RANO/SNMMI guideline, based on a [^68^Ga]Ga-DOTATOC PET/CT and considered positive if uptake was higher than liver and pituitary gland [8]. Additional requirements included an ECOG performance status of ≤ 3 and the absence of renal, hepatological, or hematological dysfunction, demonstrated by laboratory results [8].

### Angiographic intervention and [^177^Lu]Lu-DOTATATE administration

[^177^Lu]Lu-DOTATATE was administered in accordance with standard protocol for NETs [15]. The treatment plan was to complete four cycles of approximately 7.4 GBq/cycle of [^177^Lu]Lu-DOTATATE. To ensure renal protection, concomitant amino acid (lysine and arginine) was infused starting 30 min prior to radiopharmaceutical injection or at the start of the angiography, with a total infusion duration of four hours. The interventional radiologist identified the tumor feeding vessel (TFV) via femoral approach. In cases where multiple TFVs were identified, a proximal injection site was selected, covering all TFVs. Once the target artery was confirmed and the microcatheter was correctly positioned, the nuclear medicine physician administered the radiopharmaceutical, marking the procedure a technical success. Due to local radiation safety guidelines, patients were required to remain hospitalized overnight [15].

### Outcome measurements

All data were collected from clinical records; no study-specific measurements were performed for this observational study. The primary outcome measures were progression-free survival (PFS) and overall survival (OS). PFS was defined as the time from the first treatment cycle to progressive disease (PD). PD was defined according to the Proposed Response Assessment and endpoints for meningioma clinical trials, as outlined in the report from the Response Assessment in Neuro-Oncology (RANO) Working Group [16], or to significant clinical deterioration. OS was defined as the time from the first treatment cycle to death from any cause. A subgroup analysis of the PFS was made for grade 2 patients, as the largest histological grade group within the cohort.

For secondary efficacy outcomes, radiological tumor response was evaluated in accordance to RANO [16]. Objective response rate (ORR) was defined as the proportion of patients achieving complete response (CR), partial response (PR) or minor response (MR). DCR was defined as the proportion of patients achieving CR, PR, MR or stable disease (SD). Safety and toxicity were evaluated based on treatment-related adverse effects and angiographic complications. Patients were seen at the out-patient clinic for follow-up assessments in between treatment cycles, to evaluate their clinical status, including neurological deficits, ECOG performance status, and to assess adverse events according to Common Terminology Criteria for Adverse Events (CTCAE), version 5.0 [17]. In addition, laboratory tests were performed to assess liver, renal or hematologic function, also assessed according to CTCAE v5.0. Procedure-related complications were extracted from the interventional radiology reports.

### Statistical analysis

Descriptive statistics consisted of frequency analysis and are reported as medians with ranges due to the small sample size of this cohort. Survival estimates, including 95% confidence intervals (CIs), for PFS and OS were calculated by means of the Kaplan-Meier method. ORR and DCR were calculated with the Clopper-Pearson exact binominal method. Other outcomes, safety and toxicity, were reported descriptively. All statistical analyses were performed with RStudio 4.4.3.

## Results

### Patient and treatment characteristics

Seventeen patients who received their first treatment cycle between March 2018 and June 2024 were analyzed. Median follow-up duration was 36 months (range: 1–57 months), and median age at first treatment cycle was 64 years (range: 38–78 years). Histopathological grading was grade 1 in 18%, grade 2 in 59% and grade 3 in 12%. Two tumors (12%) could not be graded: one due to the absence of a pathological analysis report or specimen from surgery performed several years previously at an external center, and the other due to the lack of tumor tissue, as the patient had only received radiotherapy. Tumor location was multifocal in 47% of patients. 24% were located at the skull base, 24% at the convexities, and 6% orbital. All but one patient underwent both surgery and adjuvant radiotherapy as prior treatments (94%), with the majority having undergone multiple resections (56%) and one course of radiotherapy (71%). The most prevalent neurological deficit was visual impairment (53%). Before initiating the first treatment cycle, 41% had an ECOG performance status of 0.

Patients received a median of 3 intra-arterial cycles (range: 1–4 cycles), with 35% completing 4 intra-arterial cycles. Median administered activity per cycle was 7.5 GBq (range: 3.7–7.8 GBq). Median cumulative administered activity was 28.8 GBq (range: 7.4–30.6 GBq), and median interval between cycles was 54 days (range: 28–90 days). Main arteries selected as injection site were the external carotid artery and side branches (61%), the common carotid artery (22%) and the internal carotid artery and side branches (17%). The technical success rate was 100%. For a comprehensive overview of patient characteristics, see Table 1.

**Table 1:** Baseline characteristics

	*N* (%)
**Number of participants**	17 (100)
**Median age in years at first cycle**	64
**Sex** Male Female	6 (35) 11 (65)
**WHO grade** ^**a**^ Unknown Grade Grade 1 Grade 2 Grade 3	2 (12) 3 (18) 10 (59) 2 (12)
**Location** Multifocal Skull base Convexity Orbital	8 (47) 4 (24) 4 (24) 1 (6)
**Prior interventions** Surgery *≥ 2* *< 2* Adjuvant radiotherapy *≥ 2* *< 2* Radiotherapy monotherapy	16 (94) *9 (56)* *7 (44)* 16 (94) *4 (25)* *12 (75)* 1 (6)
**Median number IA-cycles** 4 cycles < 4 cycles	3.0 6 (35) 11 (65)
**Most prominent neurologic deficit** Vertigo Seizures Motor impairment Visual impairment Sensory impairment Cognitive impairment Gait disorders Asymptomatic	1 (6) 1 (6) 3 (18) 6 (35) 1 (6) 1 (6) 2 (12) 2 (12)
**ECOG Performance Status** 0 1 2 3	7 (41) 4 (24) 5 (29) 1 (6)
**Number of IA-procedures**	46 (100)
**Target arteries** Common carotid artery External carotid artery Internal carotid artery Occipital artery Ophthalmic artery Middle meningeal artery Internal maxillary artery	10 (22) 11 (24) 5 (11) 4 (9) 3 (7) 11 (24) 2 (4)
**Technical success rate**	100%
**Median cumulative activity in GBq (range)**	29 (7–31)
**Median time in days between cycles (range)**	54 (28–98)

WHO = World Health organization, IA = Intra-arterial, GBq = Giga Becquerel, ECOG = Eastern Cooperative Oncology Group

^a^ The classification is based on the 2021 WHO Classification of Tumors of the Central Nervous System [32]

## Survival analysis

**Fig. 1:**
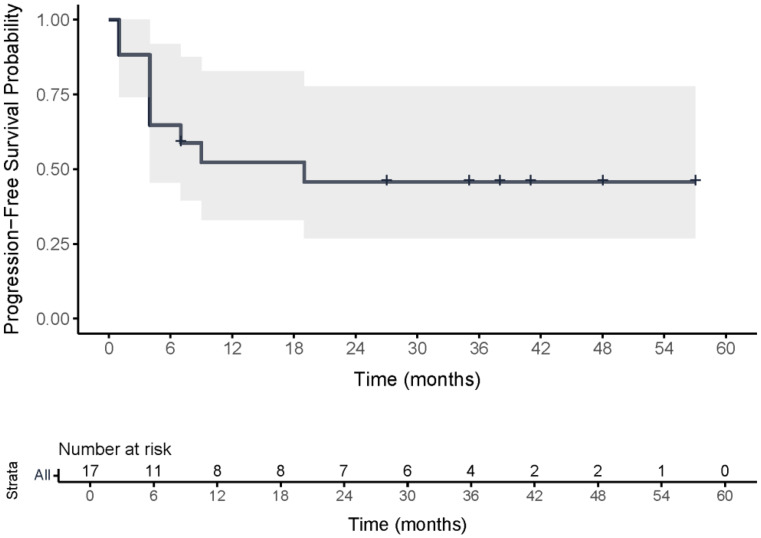
Kaplan–Meier curve of progression-free survival following intra-arterial [¹⁷⁷Lu]Lu-DOTATATE in refractory meningioma

**Fig. 2:**
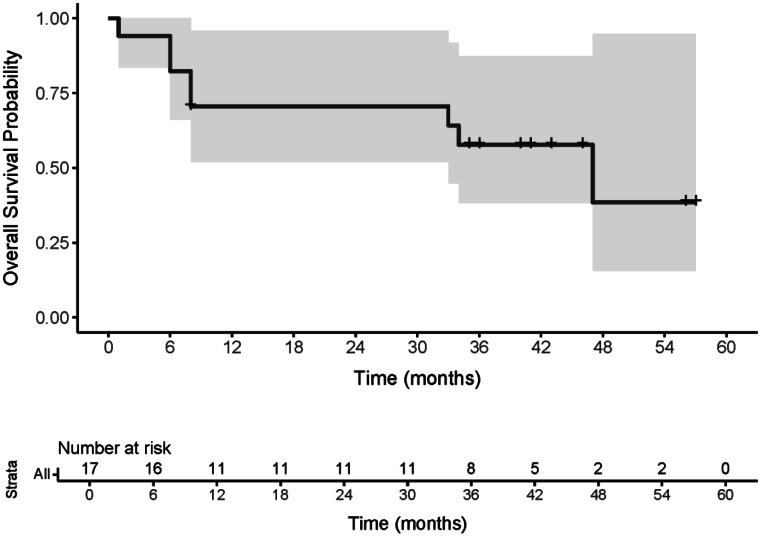
Kaplan–Meier curve of overall survival following intra-arterial [¹⁷⁷Lu]Lu-DOTATATE in refractory meningioma

PFS rates at 6, 12, and 24 months were 65% (95% CI: 45–92%), 52% (95% CI: 33–83%), and 46% (95% CI: 27–78%), respectively. OS rates were 82% (95% CI: 66–100%) at 6 months, and 71% (95% CI: 52–96%) at both 12 and 24 months. For grade 2 meningioma, PFS rates at 6,12 and 24 months were 60% (95% CI: 36–100%), 40% (95% CI: 19–86%) and 30% (95% CI: 12–77%), and OS rates were 80% (95% CI: 59–100%) at 6 months and 70% (95% CI: 47–100%) at both 12 and 24 months. Median PFS (mPFS) was 19 months (95% CI: 4–not reached) and median OS (mOS) was 47 months (95% CI: 33–not reached). For grade 2 meningiomas, mPFS was 8 months (95% CI: 4–not reached) and mOS was 41 months (95% CI: 8–not reached). PD occurred in 8 patients, 7 of whom subsequently died from the disease. One patient died from heart failure after the first cycle, considered unrelated to [^177^Lu]Lu-DOTATATE. Kaplan-Meier curves are shown in Figs. 1 and 2.

### Efficacy

ORR was 24% (95% CI: 7–50%), and DCR was 53% (95% CI: 28–77%); five patients demonstrated SD, two patients achieved PR, one patients showed MR, and one patient exhibited CR. The remaining eight patients (47%) experienced PD. One patient with multifocal disease underwent resection of a frontal meningioma after the third follow-up MRI, because of oligoprogression. However, as the patients remaining meningiomas remained stable over time, as confirmed by subsequent follow-up imaging (+ 1.5 years), this case was classified as SD.

**Fig. 3:**
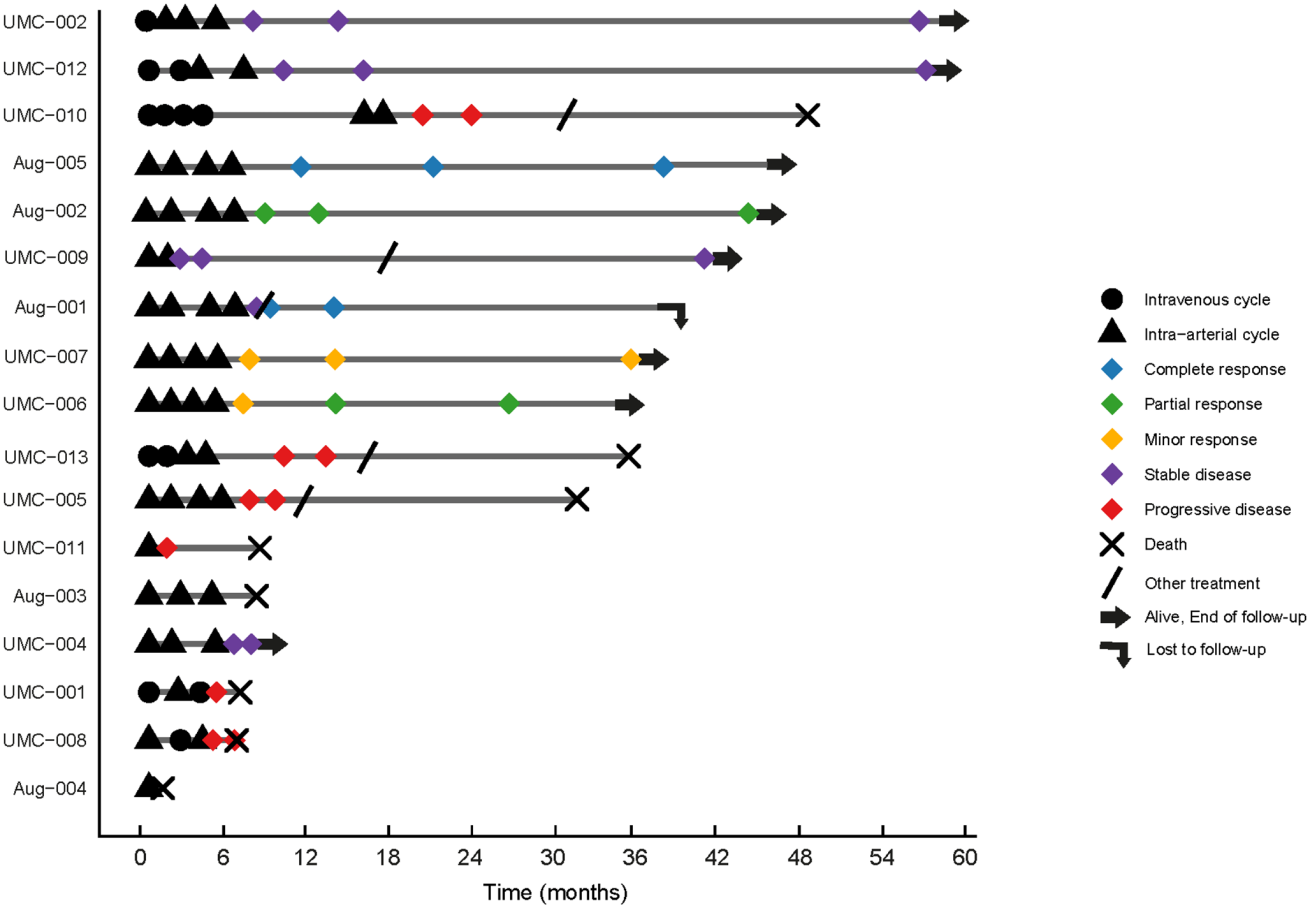
Swimmer plot of refractory meningioma patients treated with intra-arterial [^*177*^Lu]Lu-DOTATATE. Swimmer plot of patients with refractory meningioma treated with intra-arterial [¹⁷⁷Lu]Lu-DOTATATE. Each horizontal bar represents an individual patient, illustrating the follow-up period from the first treatment cycle until death or last known follow-up. Please refer to the legend for an explanation of the various symbols used. Other treatments after progression include talazoparib, panitumumab and re-resection. Subject UMC-009 underwent resection of a frontal meningioma after the third follow-up MRI showed progression. However, subsequent imaging confirmed stability of the remaining meningiomas, and the case was therefore classified as SD

During treatment, six patients experienced either progression of pre-existing neurological deficits or the development of new symptoms; four due to PD (all presenting with increase of motor impairment), one due to symptomatic radionecrosis (presenting with increased motor impairment) and one due to Stroke-like Migraine Attacks after Radiation Therapy (SMART syndrome; characterized by a transient episode of delirium). Following treatment, four patients experienced a worsening of neurological deficits (two with motor impairment and two with visual impairment), three due to PD and one due to a postoperative complication following a clinically indicated operation for a non-progressive meningioma. Neurologic deficits reported before treatment (visual impairment and hypesthesia) remained stable in two patients. One patient who was asymptomatic remained asymptomatic. Lastly, three patients experienced an improvement in neurologic symptoms, specifically a reduction in seizures and a decrease in visual impairment. See Fig. 3 and Supplemental Table 1 for a detailed depiction of the treatment trajectory.

### Safety and toxicity

Two patients exhibited a grade 3 adverse effect after treatment, namely anemia, and required transfusion. One of these patient developed a pancytopenia; however, this was not attributed to the [^177^Lu]Lu-DOTATATE treatment, but rather related to a newly discovered underlying myelodysplastic syndrome (MDS), due to chronic myeloid leukemia and allogeneic stem cell transplantation. One patient required a 50% dose reduction of [^177^Lu]Lu-DOTATATE during a single treatment cycle due to a leucopenia grade 2. Two patients had discontinued their treatment: one due to symptomatic radionecrosis, and the other as a result of delirium secondary to SMART syndrome.

**Fig. 4:**
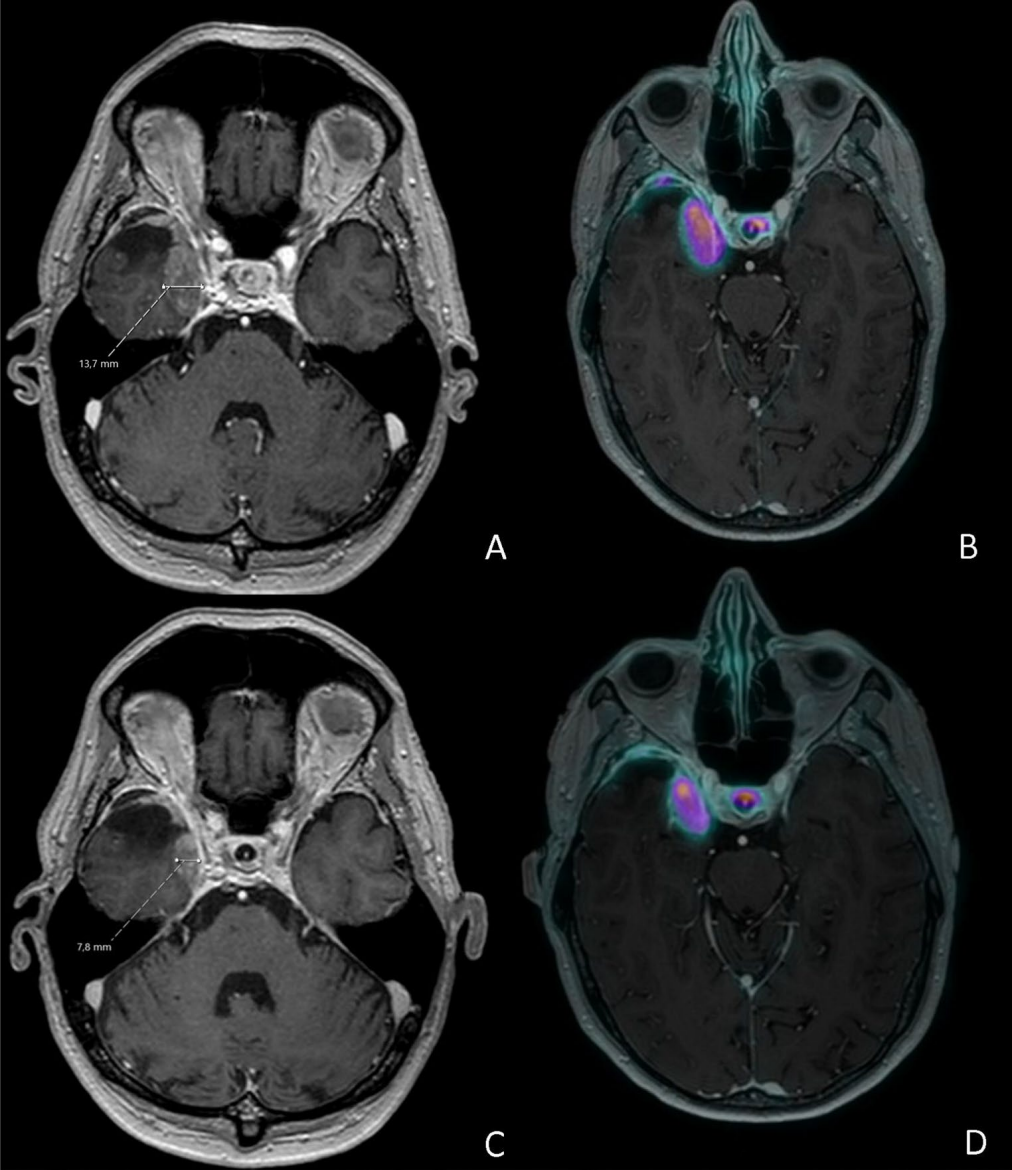
Case example (UMC-006). **A** Pre-treatment gadolinium-enhanced T1-weighted MRI showing a meningioma in the right sphenoid cavity with a maximum horizontal diameter of 13.8 mm. **B** Pre-treatment [⁶⁸Ga]Ga-DOTATOC PET/CT overlaid on MRI demonstrating high somatostatin receptor expression in the lesion. **C** Post-treatment gadolinium-enhanced T1-weighted MRI showing an 83% volume reduction, consistent with a partial response. **D** Post-treatment [⁶⁸Ga]Ga-DOTATOC PET/CT overlaid on MRI demonstrating decreased somatostatin receptor expression

The angiographic intervention was generally safe and well tolerated (case example shown in Fig. 4), with only one patient experiencing a potentially procedure-related complication, namely a local infection and necrosis of a finger. Two patients experienced adverse effects related to the amino acid infusion administered for renal protection, specifically hyperkalemia, headache and nausea (grade 1). Supplemental Table 1 outlines relevant clinical characteristics and most severe adverse events .

## Discussion

Meningiomas are associated with some of the highest survival rates among intracranial tumors. However, a progressive and treatment-refractory subset lacks an effective, evidence-based therapeutic option that offers durable (progression-free) survival. The present multicenter cohort study confirms safety of intra-arterial [^177^Lu]Lu-DOTATATE and suggests improved survival outcomes, compared to the benchmark [6]. Our real-world cohort forms a valid representation of the population of treatment-refractory meningiomas, illustrated by the multiple lines of local therapy (surgery and/or EBRT) that they had received.

In these 17 patients, the PFS6 rate was 65% overall and 60% for grade 2 meningioma, comparing favorably to recently published benchmarks for recurrent meningioma. Kotecha et al. reported benchmark PFS6 rates of 67% for WHO grade 1 and 49% for grades 2–3. Targeted therapies demonstrated the highest efficacy in WHO grade 1 meningiomas, with a pooled PFS6 of 62%, while immunotherapy achieved a pooled PFS6 of 46% in WHO grade 2 and 3 meningiomas [6]. Their meta-analysis included two [^177^Lu]Lu-DOTATATE studies, as they were the only ones with extractable data [18, 19]. Notably, the PFS6 rate of 65% in the present cohort approaches the benchmark reported for WHO grade 1 meningiomas, despite the fact that only 3 of 17 patients had grade 1 disease.

Most studies evaluating the efficacy of [^177^Lu]Lu-DOTATATE have primarily utilized intravenous administration as route of delivery. In a meta-analysis conducted by Mirian et al., six studies encompassing a total of 111 patients were analyzed. They reported a PFS6 of 61% and a 12-PFS of 53%, slightly lower compared to the present study. Furthermore, the DCR reported was 59% and ORR was only 2%, the latter substantially lower than 24% ORR observed in the present cohort [9]. Other retrospective studies that were published subsequently reported comparable findings to Mirian et al., with PFS6 ranging from 43 to 61% [10, 20–22]. The largest prospective study to date, conducted by Severi et al. included 37 patients treated with [^177^Lu]Lu-DOTATATE and demonstrated a DCR of 54% and a mPFS of 15 months [11]. Only Salgues et al. reported a higher PFS6 of 85.7%, including only seven patients. Notably their ORR was 0% [18]. Hartampf et al. reported a remarkably high mPFS of 91 months; however, it is important to consider that 60% of their cohort had grade 1 meningioma at the start of treatment and were not classified as treatment-refractory. These patients remained eligible for, and received, EBRT in conjunction with [^177^Lu]Lu-DOTATATE, potentially conferring a more favorable baseline prognosis [23].

Two retrospective cohort studies have reported on the efficacy and safety of intra-arterial administration of [^177^Lu]Lu-DOTATATE. In the study by Puranik et al., 8 patients were included, four of whom received intra-arterial [^177^Lu]Lu-DOTATATE [24]. The study had a follow-up period of 12 months and reported a mPFS of 8.9 months. A DCR of 75% and ORR of 25% were observed; however, these outcomes were assessed only four weeks after the second treatment cycle. At the 12-month follow-up, only one patient maintained SD. Notably, only two patients completed all four treatment cycles, and all patients received their first cycle via intravenous administration [24]. Amerein et al. included 13 patients and reported a 6- and 12-PFS rates of 77%, DCR 77% and ORR of 15%, which exceeded the current study results, except for the ORR [25]. However, four patients in the cohort by Amerein et al. received [^177^Lu]Lu-DOTATATE as a first-line treatment and another four patients had only undergone surgery prior to [^177^Lu]Lu-DOTATATE. In addition, the relative low proportion of grade 2–3 tumors (31%) further contributed to a more favorable baseline profile compared to the current presented treatment-refractory cohort [25].

This study demonstrated that intra-arterial [^177^Lu]Lu-DOTATATE is generally a well-tolerated therapy, and that, when performed by experienced practitioners, angiographic risk remains limited (in this cohort a single complication: local necrosis). Studies evaluating the safety of [^177^Lu]Lu-DOTATATE have demonstrated a favorable toxicity profile, with grade 3–4 adverse events being rare, typically reversible, and infrequently progressing to chronic conditions. It can be hypothesized that intra-arterial administration may be associated with a higher risk of adverse effects due to the anticipated increase in tumor absorbed dose. However, in the present study, only two patients experienced grade 3 toxicity (12%), specifically anemia requiring transfusion, a rate comparable to phase 3 NET trials [15, 26]. One of the grade 3 anemias was associated with the development of low-risk MDS, diagnosed within one year after last treatment cycle. This patient had a medical history of chronic myeloid leukemia, for which he underwent allogeneic stem cell transplantation and high-dose cyclophosphamide. While MDS has been a rare complication of [^177^Lu]Lu-DOTATATE in NET patients (1–2%) [27, 28], no data are currently available regarding its occurrence in patients with a history of allogeneic stem cell transplantation. Prior use of alkylating agents, such as cyclophosphamide, is a recognized risk factor for myelotoxicity after [^177^Lu]Lu-DOTATATE [29]. Two patients were unable to continue treatment due to increasing frailty; one as a result of symptomatic radionecrosis and the other due to SMART syndrome. Initially both cases were suspected to represent PD; however, this was ruled out based on the absence of radiological evidence of tumor progression on MRI. The patient with SMART syndrome recovered without medical intervention and has remained progression-free after 41 months. The patient with symptomatic radionecrosis responded well to treatment with dexamethasone and bevacizumab. Both SMART syndrome and radionecrosis are known complications of prior radiotherapy, which both patients have received [30, 31]. Currently, evidence is insufficient to support an association between these complications and PRRT.

Several limitations should be acknowledged. First, the retrospective design inherently introduces the potential for referral and selection biases. Additionally, the reliance on retrospective data makes the study dependent on the accuracy of physician documentation in patient records, i.e. reporting bias. The participation of two centers located in different countries may have introduced variability in reporting practices and clinical protocols. Moreover, post-treatment tumor dosimetry was not available, limiting the ability to accurately assess the extent of dose absorption by the tumor. Another limitation is the absence of a control group, which restricts the ability to draw definitive conclusions regarding treatment efficacy. The cohort size, although large in comparison to previous studies in this setting, is too small to identify specific clinical or histomolecular subgroups that are most likely to benefit from this treatment. Notably, the European Organization for Research and Treatment of Cancer has initiated a large multinational randomized controlled trial (EORTC-2334-BTG LUMEN-1) to evaluate the impact of intravenous [^177^Lu]Lu-DOTATATE on PFS in patients with recurrent meningioma. This presents the possibility of conducting a separate or parallel prospective trial to investigate intra-arterial administration and allow for direct comparison.

This study supports that intra-arterial [^177^Lu]Lu-DOTATATE monotherapy is a safe treatment option, surpassing established survival benchmarks for systemic therapies in refractory meningioma trials and demonstrating a higher ORR than historical data on intravenous administration. These results, however, require validation through a prospective, controlled study to strengthen the evidence base, confirm the observed outcomes, and determine which histomolecular or clinical subgroups are most likely to obtain benefit.

## Supplementary Information

Below is the link to the electronic supplementary material.

Supplementary Material 1

## Data Availability

All relevant data supporting the findings of the current study are provided in Supplemental Table 1.
