# Supplementary material for: Efficacy of Intra-arterial [177Lu]Lu-DOTATATE monotherapy for treatment-refractory meningioma
Source: J Neurooncol. 2025 Oct 15;176(1):8. doi: 10.1007/s11060-025-05266-9 (PMC12528330; doi:10.1007/s11060-025-05266-9)
Supplement: Supplementary file 1 — Supplementary Material 1 [file 11060_2025_5266_MOESM1_ESM.docx]

| **Supplemental Table 1. Comprehensive Overview of Patient Demographics, Tumor Characteristics, Interventions, and Treatment Outcomes** | | | | | | | | | | |
| --- | --- | --- | --- | --- | --- | --- | --- | --- | --- | --- |
| ***Patient*** | ***WHO grade ^a^*** | ***Gender*** | ***Age ^b^*** | ***Location*** | ***Prior interventions*** | ***Total # cycles***  ***(# IA cycles)*** | ***Cumulative activity (MBq)*** | ***Best radiologic response ^c^*** | ***Most severe adverse effect*** | ***Neurological deficit*** |
| UMC-001 | 2 | Female | 66 | Multifocal | 2 surgeries & 6 radiation treatments | 3 (1) | 22585 | PD | - | Vertigo |
| UMC-002 | 2 | Female | 62 | Convexity | 2 surgeries & 1 radiation treatment | 4 (3) | 29511 | SD | Alopecia (grade 1) | Epilepsy |
| UMC-004 | 1 | Male | 71 | Multifocal | 3 surgeries & 1 radiation treatment | 3 (3) | 22356 | SD | Thrombocytopenia (grade 2) | Motor impairment |
| UMC-005 | 2 | Female | 38 | Multifocal | 3 surgeries & 1 radiation treatment | 4 (4) | 30672 | PD | Anemia (grade 1) | Visual impairment |
| UMC-006 | 1 | Male | 58 | Skull base | 1 surgery & 1 radiation treatment | 4 (4) | 30510 | PR | Anemia (grade 3) ^d^ | Asymptomatic |
| UMC-007 | 1 | Female | 71 | Skull base | 1 surgery & 1 radiation treatment | 4 (4) | 29952 | MR | Alopecia (grade 1) | Hypoesthesia |
| UMC-008 | 3 | Female | 76 | Multifocal | 2 surgeries & 1 radiation treatment | 3 (2) | 22494 | PD | Thrombocytopenia (grade 1) | Gait disorder |
| UMC-009 | 2 | Female | 61 | Multifocal | 4 surgeries & 2 radiation treatments | 2 (2) | 15003 | SD | Fatigue (grade 1) | Visual impairment |
| UMC-010 | 2 | Female | 58 | Orbital | 2 surgeries & 1 radiation treatment | 6 (2) | 29766 | PD | Chemosis | Visual impairment |
| UMC-011 | 2 | Male | 72 | Multifocal | 1 surgery & 1 radiation treatment | 1 (1) | 7407 | PD | Fatigue (grade 1) | Cognitive impairment |
| UMC-012 | 0 | Female | 46 | Multifocal | 2 surgeries & 2 radiation treatments | 4 (2) | 25751 | SD | Lymphocytopenia (grade 2) | Visual impairment |
| UMC-013 | 2 | Male | 57 | Skull base | 2 surgeries & 2 radiation treatments | 4 (2) | 28856 | PD | Fatigue (grade 1) | Asymptomatic |
| Aug-001 | 2 | Male | 78 | Convexity | 1 surgery & 1 radiation treatment | 4 (4) | 29851 | SD | Anemia (grade 3) | Motor impairment |
| Aug-002 | 2 | Male | 73 | Convexity | 1 surgery & 1 radiation treatment | 4 (4) | 29685 | PR | Lymphocytopenia (grade 1) | Gait disorder |
| Aug-003 | 3 | Female | 64 | Convexity | 1 surgery & 1 radiation treatment | 3 (3) | 22280 | PD | - | Motor impairment |
| Aug-004 | 2 | Female | 67 | Multifocal | 1 surgery & 1 radiation treatment | 1 (1) | 7457 | PD | Local necrosis ^e^ | Motor impairment |
| Aug-005 | 0 | Female | 59 | Skull base | 1 radiation treatment | 4 (4) | 30024 | CR | - | Visual impairment |
| Legend: WHO = World Health organization, IA = Intra-arterial, MBq = Mega Becquerel, CR = complete response, PR = partial response, MR = minor response, PD = progressive disease, SD = stable disease  ^a^ The grading is based on the 2021 WHO Classification of Tumors of the Central Nervous System [32]  ^b^ Age at first treatment cycle  ^c^ Based on the Response Assessment in Neuro-Oncology (RANO) Working Group [16]  ^d^ This patient had a medical history of chronic myeloid leukemia, for which he underwent allogeneic stem cell transplantation and high-dose cyclophosphamide  ^e^ Complication of angiographic intervention | | | | | | | | | | |
